# Emotions Modulate Affordances-Related Motor Responses: A Priming Experiment

**DOI:** 10.3389/fpsyg.2022.701714

**Published:** 2022-06-08

**Authors:** Flora Giocondo, Anna M. Borghi, Gianluca Baldassarre, Daniele Caligiore

**Affiliations:** ^1^Laboratory of Embodied Natural and Artificial Intelligence, Institute of Cognitive Sciences and Technologies, National Research Council, Rome, Italy; ^2^Department of Dynamic and Clinical Psychology, Sapienza University of Rome, Rome, Italy; ^3^Institute of Cognitive Sciences and Technologies, National Research Council, Rome, Italy; ^4^AI2Life s.r.l., Innovative Start-up, ISTC-CNR Spin-off, Rome, Italy; ^5^Computational and Translational Neuroscience Laboratory, Institute of Cognitive Sciences and Technologies, National Research Council, Rome, Italy

**Keywords:** affordances, approach motivational state, aversive motivational state, emotions, dangerous objects, motor responses, network neuroscience, neutral objects

## Abstract

Traditionally, research on affordances and emotions follows two separate routes. For the first time, this article explicitly links the two phenomena by investigating whether, in a discrimination task (artifact vs. natural object), the motivational states induced by emotional images can modulate affordances-related motor response elicited by dangerous and neutral graspable objects. The results show faster RTs: (i) for both neutral and dangerous objects with neutral images; (ii) for dangerous objects with pleasant images; (iii) for neutral objects with unpleasant images. Overall, these data support a significant effect of emotions on affordances. The article also proposes a brain neural network underlying emotions and affordance interplay.

## Introduction

Gibson (1979) used the term “affordance” earliest to indicate the potential actions elicited by the observation of objects in the environment. The orientation of object graspable parts (Tucker and Ellis, 1998), the object size and graspability (Anelli et al., 2013a), the perceived distance of an object (Mustile et al., 2021), or its harmfulness (Anelli et al., 2012a) are all aspects influencing affordances.

For the first time, this article investigates the potential connection between affordances and emotions. We consider emotions as action readiness that can be driven by approach or aversive motivational states (Lang, 1995). Few studies investigated how the observers’ emotions can modulate their interactions with objects that can be emotionally charged, such as dangerous objects (i.e., knife). For example, studies have shown that safe/neutral objects evoke an affordance effect (approach motivational state aimed to interact with the object) while dangerous objects evoke an interference/inhibitory effect (aversive motivational state aimed to avoid the object). This motor response is reflected in faster reaction times (RTs) for neutral objects than for dangerous objects (Anelli et al., 2012a, 2013a). Although interesting, these studies investigate the relationship between affordances and emotions only indirectly. Here, we address it directly, proposing an experiment with emotional images as prime. In particular, we intend to demonstrate how the affordance-related motor responses change, through an emotional prime, that should induce an approach and aversive motivational states. More specifically, we expect that an emotional prime, such as a pleasant or an unpleasant image, worsens the affordance-related motor responses, whereas a neutral image should improve the perception of affordances.

Here, we propose an experiment using emotional images as primes. Our study explicitly addresses how the approach and aversive motivational states affect affordances-related motor responses. In particular, through a categorization task (artifact vs. neutral objects), we investigate whether motivational states induced by emotional images: (i) modulate the motor responses; (ii) influence the perception of the objects’ dangerousness (a feature totally irrelevant to the task). To address these two issues, we measure response time (RT), the time that elapses between the presentation of the stimulus and the response given.

Emotion and attention are related to one another because they both deal with information processing priorities (Oatley and Johnson-laird, 1987, for a review about emotion and attention see Dolcos et al., 2020). Attention and emotions toward an object are useful to avoid those objects that can be dangerous for the organism. A study conducted by Anderson and Phelps (2001) demonstrated the influence of emotion on attention. The authors found that it is difficult to detect a second target within a series of stimuli if the second target follows too closely the first one (e.g., Raymond et al., 1992). Crucially, participants were more likely to detect the second target if it was emotionally charged, and such effect was strongest with shorter lags between the first and second target, when the second target was usually most difficult to detect (Anderson and Phelps, 2001). Murphy et al. (2012) found that an irrelevant object potentiates action only if it receives sufficient attention. Zhao (2016) found that the affordance of dangerous objects is also sensitive to the perceptual load. An irrelevant dangerous object cannot potentiate an action if it receives insufficient attention. On this basis, we hypothesised that motivational states induced by emotional prime with pleasant and unpleasant images contribute to increasing the cognitive load thus preventing the subsequent orienting of attention. This determines a slower motor response to object affordances with respect to those obtained by using neutral images as prime. We also hypothesised that the pleasant and unpleasant emotional images influence the affordances evoked by dangerous and neutral objects. In particular, the ability of pleasant and unpleasant images to capture, narrow, and hold attention gives rise to slower RTs for neutral and dangerous objects compared to neutral and dangerous objects with a neutral emotional image. We also hypothesized that the pleasant and unpleasant emotional images influence the affordances evoked by dangerous and neutral objects. In particular, the ability of pleasant and unpleasant images to capture, narrow, and hold attention give rise to slower RTs for neutral and dangerous objects compared to neutral and dangerous objects with a neutral emotional image.

## Materials and Methods

### Participants

The experiment was attended by a total of 40 subjects (12 females and 28 males; mean age: 26.45 years; range: 19–39), Italian students of Psychology and the Advanced School of Artificial Intelligence. All of them were right-handed and had normal or corrected-to-normal visual acuity. We tested manual dominance through the Edinburgh Handedness Inventory test (Oldfield, 1971). The choice of sample size was informed by the sample sizes of similar published work (Anelli et al., 2012a). The posterior power analyses confirmed that the present sample size was reasonably close to that determined based on those analyses (see Limitation section). The Ethics Committee of the National Research Council of Italy approved the procedures, and participants gave written informed consent before starting the experiment.

### Apparatus and Stimuli

Participants sat in front of a 15.3″ colour monitor. E-Prime 2.0 software was used for presenting stimuli, collecting responses, and measuring the RTs. The experimental stimuli consisted of images of everyday graspable objects and emotional pictures selected from the International Affective Picture System-IAPS (Lang et al., 2008). The common graspable objects were displayed in 16 colour images showing artifacts and natural objects, half dangerous and half neutral (see Table 1); the dangerousness is valued as a degree of risk for pain. The stimuli size was compatible with power or precision grip. These graspable stimuli had already been used in other studies (for detailed information, see Anelli et al., 2012a,b, 2013b).

**Table 1:** The 16 graspable objects.

	Dangerous objects	Neutral objects
Natural objects	Cactus	Cat
	Husk	Chick
	Porcupine	Plant
	Scorpio	Tomato
Artifact objects	Broken bulb	Bulb
	Broken glass	Glass
	Knife	Lighted out match
	Lighted match	Spoon

The 51 experimental emotional images^1^ aimed to induce a motivational state were selected from IAPS. The IAPS is a database of pictures designed to provide a standardised set of images for studying emotions. In particular, IAPS images are divided into unpleasant images that should induce an aversive motivational state, pleasant images that should elicit an approach motivational state, and neutral images. We have selected 17 pleasant (mean valence/arousal 6.73/6.49), 17 neutral (mean valence/arousal 5/3.56), and 17 unpleasant (mean valence/arousal 1.92/6.30) pictures. The neutral images depicted faces, while pleasant and unpleasant images depicted sex and violent scenes, respectively. We selected the pictures from an initial set of 91 images^2^ (30 pleasant: mean valence/arousal 6.77/6.5; 28 neutral: mean valence/arousal 5.05/3.4; 33 unpleasant: mean valence/arousal 1.87/6.33), tested on a different group of 25 participants through an online survey. The survey was used to control whether any object used in the study appeared in the IAPS images in order to eliminate the semantic relationship between IAPS pictures and graspable stimuli. Participants were asked whether natural, artifact, or “no objects” appeared in each image. Moreover, they were required to write down the name of the object they saw. The percentage of “no objects” was greater than 50% of cases in the selected images. In any case, no object used in graspable stimuli appears in the IAPS images.

### Procedure

The experiment consisted of three experimental blocks: a *block with IAPS images*, *a block without IAPS images*, and a *practice block*. Across the three blocks, participants were required to perform a categorization task, deciding whether the graspable stimulus was an artifact or a natural object (1st and 2nd horizontal line of Table 1), so the Object Dangerousness (i.e., dangerous vs. neutral, left and right side of Table 1) was totally irrelevant to the task. The participants were asked to categorize each object as soon as it appeared by pressing either one of two designed keys with their left or right index finger. The use of two response keys responses and the RT as a single metric for the study of affordance is quite common in the literature (see Anelli et al., 2012a, 2013a; Zhao, 2017, 2019. We manipulated the *Response Key*: half participants had to press the “S” key for artifact objects and the “L” key for natural objects; the other half of the group had reverse instructions. Furthermore, the counterbalancing of the response keys across participants and the submission of the Edinburgh Handedness Inventory test (Oldfield, 1971) reduces the possibility that other factors beyond the tested ones influence the response time.

The *practice block* consisted of six trials; three with the emotional prime and three without emotional prime. Each trial began with a fixation cross (500 ms) displayed at the screen centre. Then an IAPS picture (2000 ms) or a graspable object (until a response had been made or 2000 ms had elapsed) was shown.

**Figure 1:**
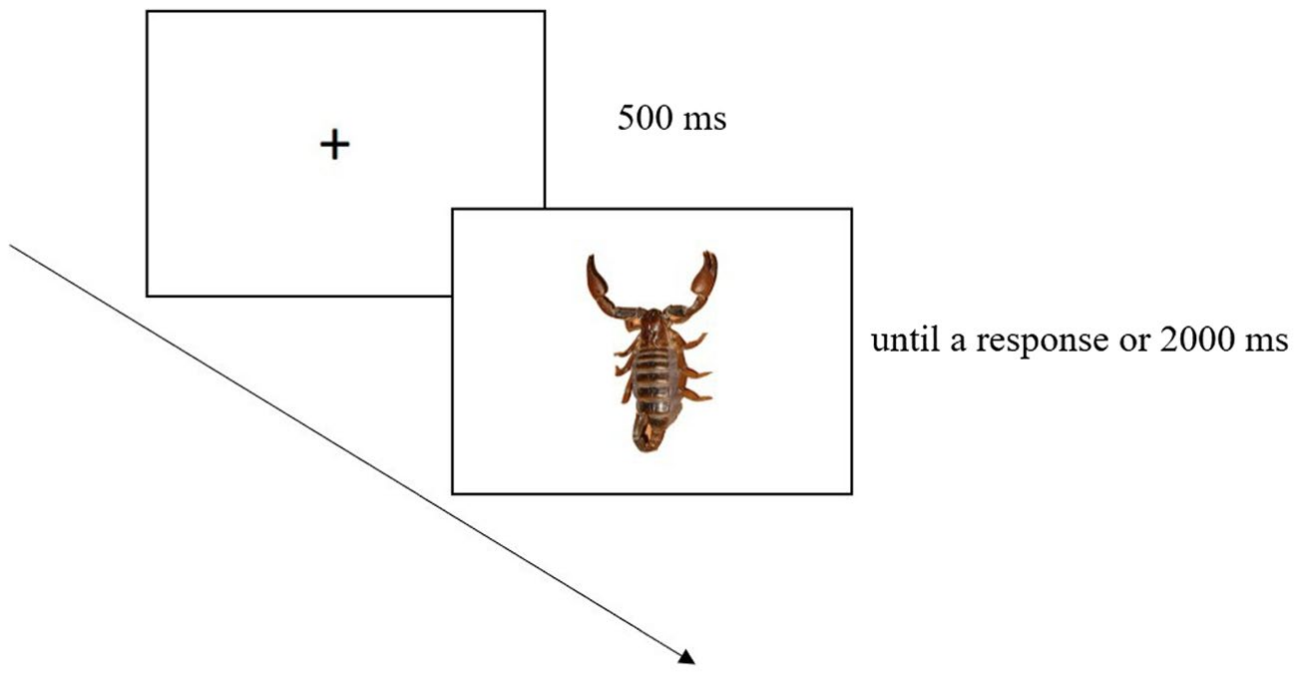
Example of trial in the *block without IAPS images*. This involved a fixation cross followed by a graspable object.

The *block without IAPS images* was made up of 96 trials; each trial began with a fixation cross (500 ms) displayed at the screen centre. Then a graspable object was shown until a response had been made or 2000 ms had elapsed. Each graspable object was presented six times (Figure 1).

**Figure 2:**
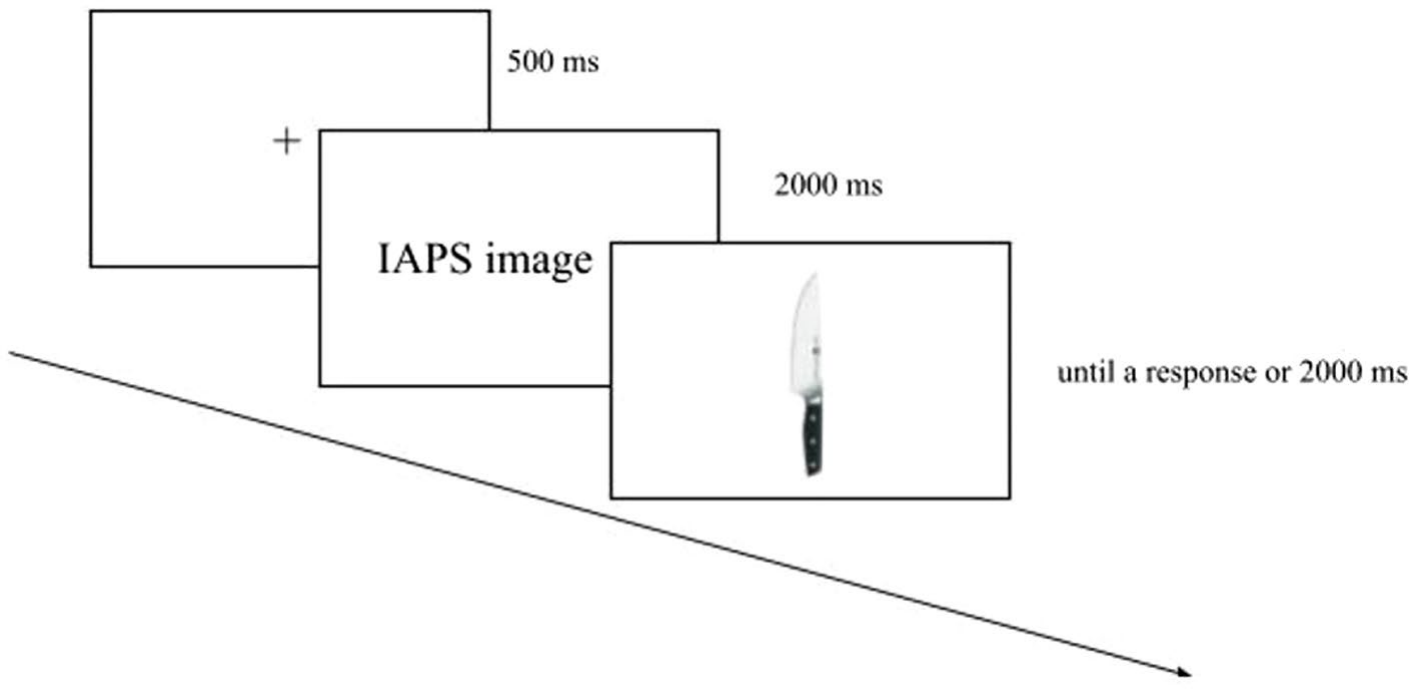
Example of trial in the *block with IAPS images*. This involved a fixation cross, followed first by the IAPS picture and then by a graspable object.

The *block with IAPS images* was made up of 48 trials consisting of an everyday graspable object preceded by an IAPS picture. Each graspable object was presented three times, always with a different IAPS image. Each trial began with a fixation cross (500 ms) displayed at the screen centre. Soon after, the IAPS picture was shown (2000 ms), and then the graspable object was shown until a response had been made or 2000 ms had elapsed (Figure 2).

The timing to present IAPS images is based on previous evidence showing that emotional displays as primes can determine subliminal priming effects (S. T. Murphy and Zajonc, 1993). Lu et al. (2011) demonstrated that response to emotional prime could be present after 20 ms. The study analysed the subliminal affective priming by recording event-related potential (ERP) to ambiguous neutral faces preceded by 20 ms positive or negative prime faces.

The experiment started with the instruction followed by a *practice block*, after the *block with IAPS images* or the *block without IAPS images*.

**Figure 3:**
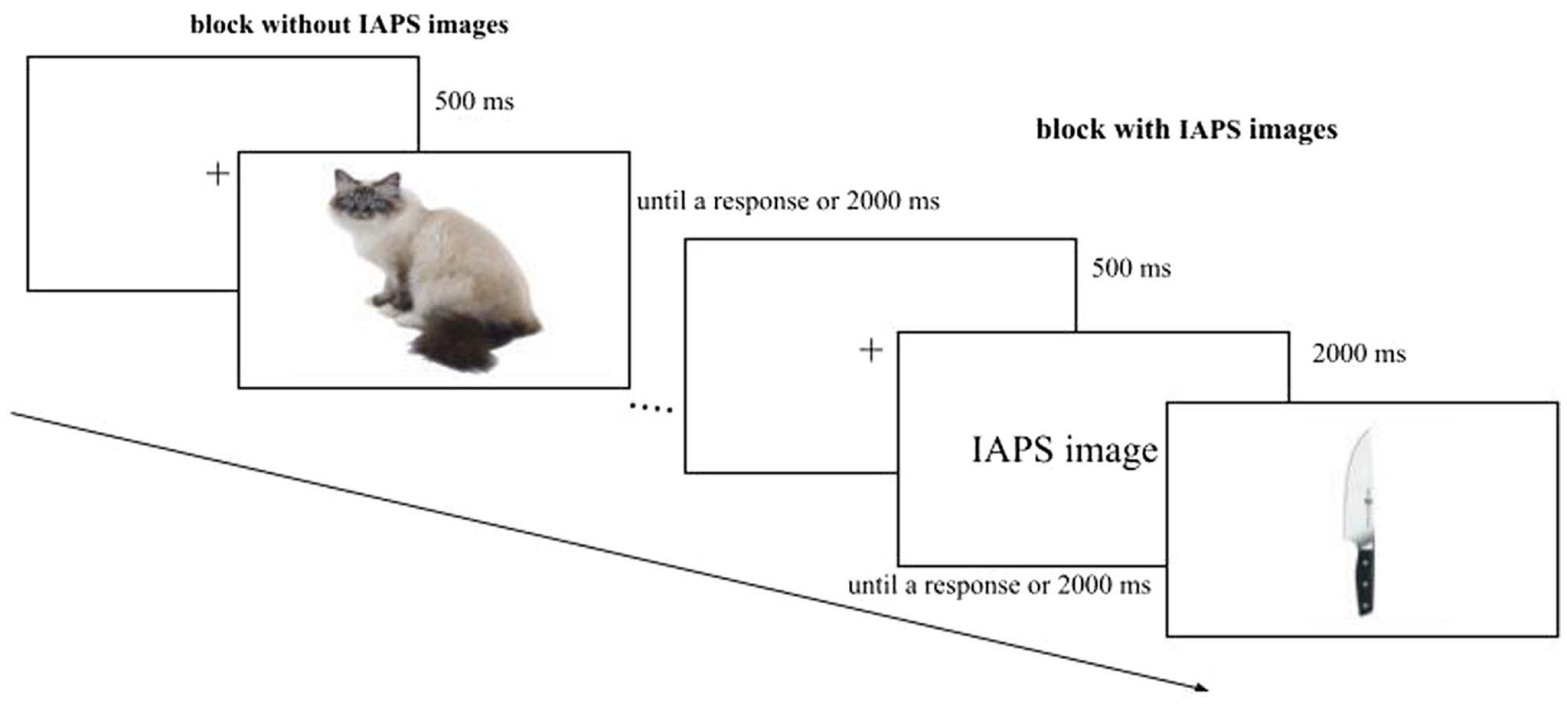
Example of the experimental paradigm. In this graphical explanation, the *block without IAPS images* is the first block to be shown, followed by the *block with IAPS images*. In the experiment, the presentation of the blocks was counterbalanced.

We had four *Sessions* where the presentation of the blocks (*with* and *without IAPS images*) and the *Response Key* (left, right) were counterbalanced. The stimuli were randomized within the block, and the order was the same for each participant (Figure 3). The data were collected in a single session. Overall the experiment consisted of 144 trials and lasted about 10 min.

### Data Analysis

The analysis excluded RTs for incorrect responses and RTs higher than two standard deviations from each participant’s overall mean (177 RTs for the *block without IAPS images* and 176 RTs for the *block with IAPS images*). The trials of the *practice block* were not included in the analysis.

We conducted two different ANOVAs: one for the *block without IAPS images* and the other one for the *block with IAPS images*. Bonferroni *post hoc* tests were conducted on significant interactions.

#### Block Without IAPS Images

The measured RTs were entered into a mixed 2 × 2 × 2 × 2 ANOVA, with *Object Dangerousness* (dangerous and neutral) and *Object Category* (artifact and natural) as within-subjects factors, and *Response Key* (the S″ key for artifact object and the “L” key for natural object, and vice versa) and *Session* (the *block without IAPS images* followed by the *block with IAPS images* or the *block with IAPS images* followed by the *block without IAPS images*) as between-subjects factors.

#### Block With IAPS Images

The measured RTs were entered into a mixed 2 × 3 × 2 × 2 × 2 ANOVA, with *Object Dangerousness* (dangerous and neutral), *IAPS Type* (pleasant, unpleasant, and neutral), and *Object Category* (artifact and natural) as within-subjects factors, and *Response Key* (the “S” key for artifact object and the “L” key for natural object, and vice versa) and *Session* (the *block with IAPS images* followed by the *block without IAPS images* or the *block without IAPS images* followed by the *block with IAPS images*) as between-subjects factors.

## Results

### Block Without IAPS Images

**Figure 4:**
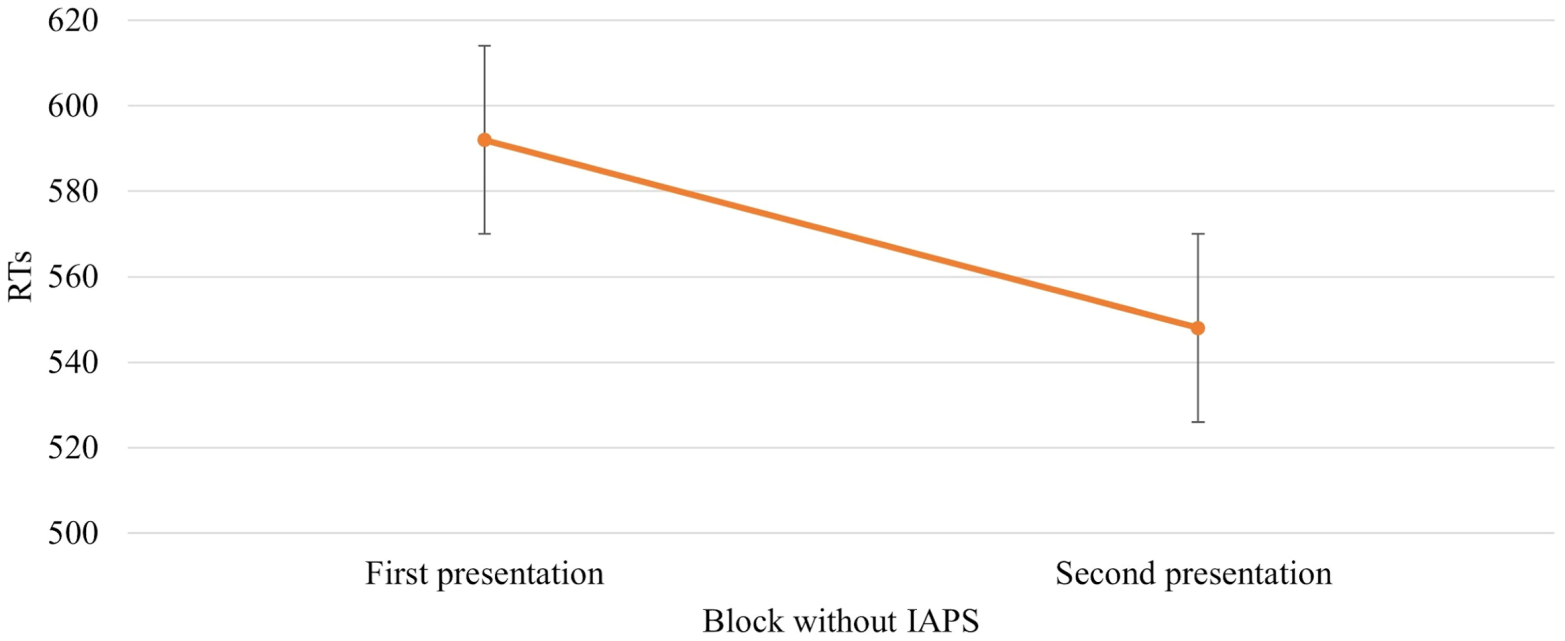
Significant *Session* effect in the *block without IAPS*. Values are in milliseconds and bars represent the standard error. “First presentation” indicates that the block without IAPS images is presented before the block with IAPS images, whereas “Second presentation” indicates the opposite situation where the block without IAPS images is presented just after the block with IAPS images.

The ANOVA revealed that the main effect of *Session* [*F* (1,672) = 39,766, *p* = 0,000, power = 1] was significant. In particular, RTs were faster when the *block without IAPS images* was the second block presented (592 vs. 548 ms, respectively; Figure 4).

**Figure 5:**
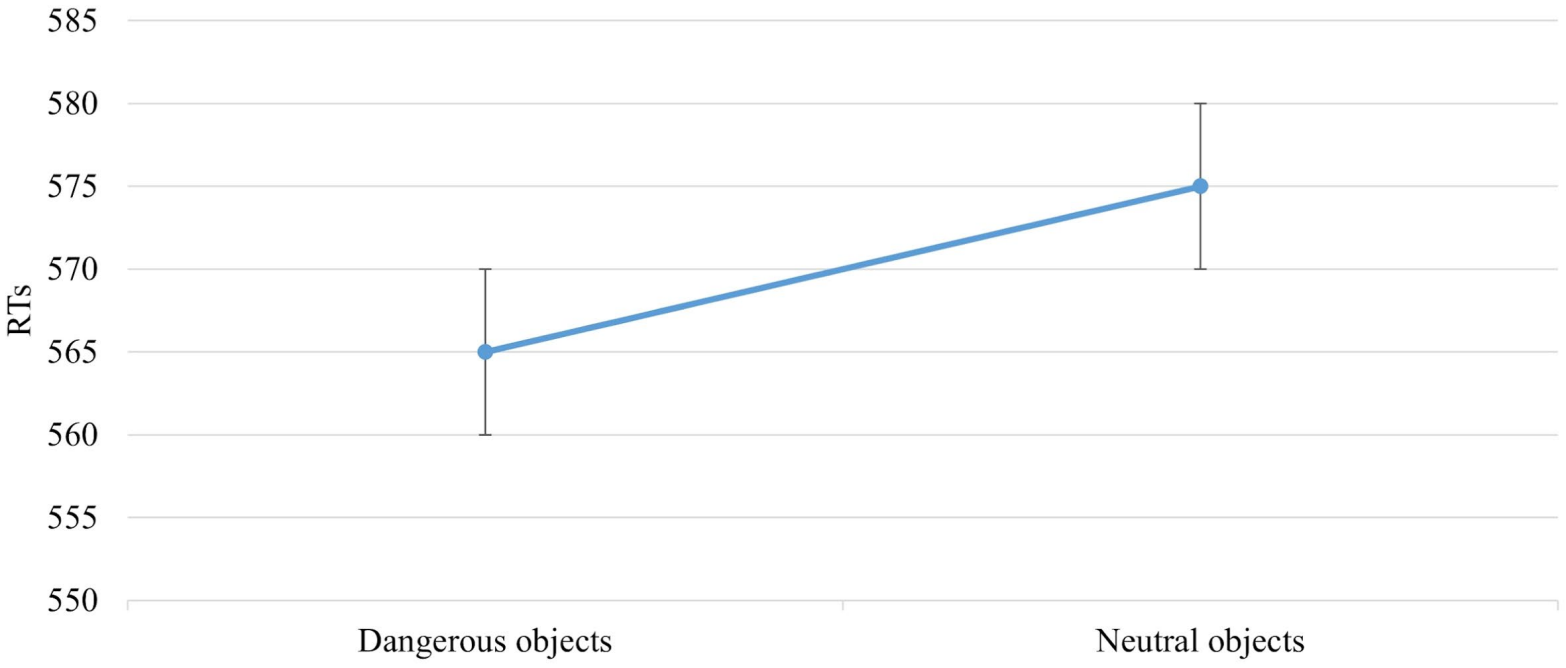
The significant main effect of *Object Dangerousness* in the *block without IAPS*. Values are in milliseconds and bars represent the standard error.

The main effect of *Object Dangerousness* [F (1,672) = 5,998, p = 0,015, power = 0,686] was significant. The *post hoc* test showed that RTs were faster when the object was dangerous than neutral (565 vs. 575 ms; Figure 5).

The interaction between *Object Category* and *Response Key* [F (1,672) = 4,857, *p* = 0,028, power = 0,595] was significant. The *post hoc* test showed that when participants had to press the “S” key for artifact and the “L” key for natural objects, the responses were faster for natural objects (571 vs. 578 ms). By contrast, when participants had to press the “S” key for natural and the “L” key for artifact objects, the responses were faster for artifact objects (560 vs. 571 ms).

No other main effect or interaction was present.

### Block With IAPS Images

**Figure 6:**
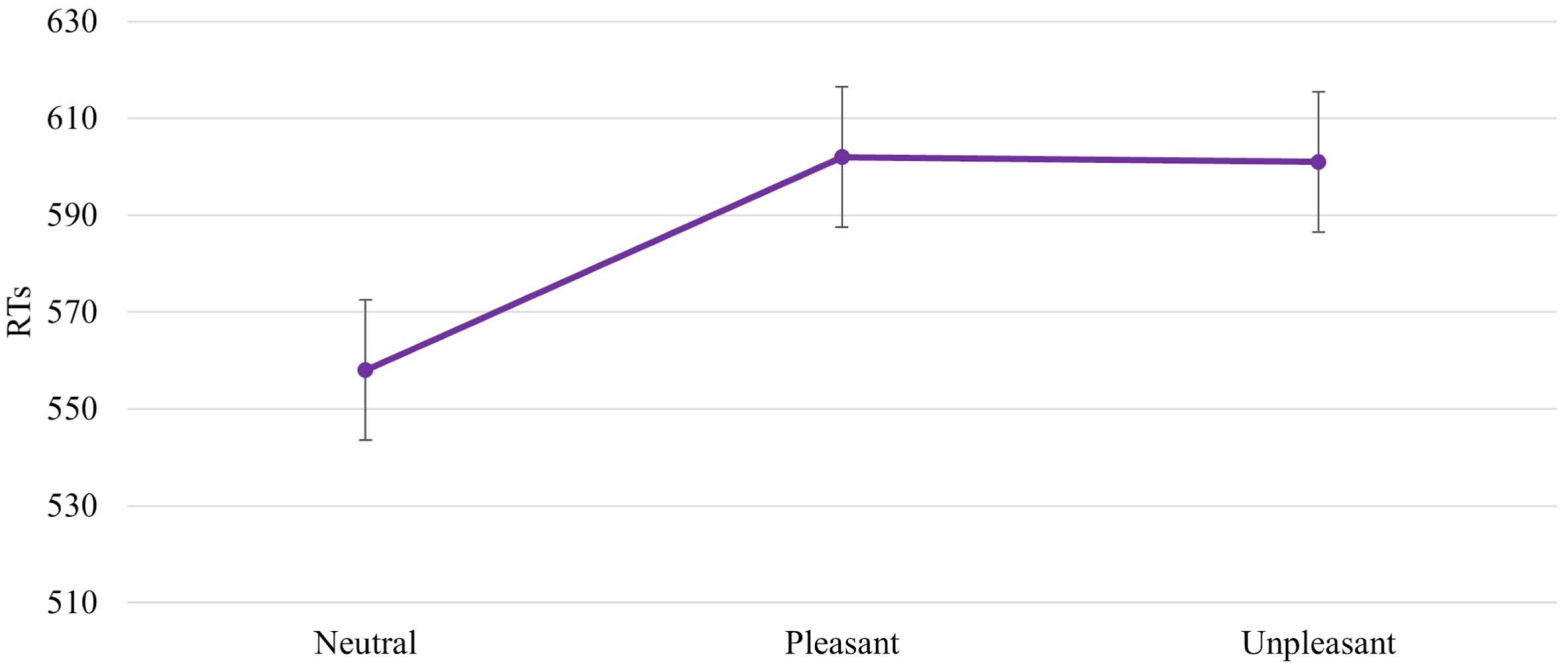
Significant *IAPS Type* effect. Values are in milliseconds and bars represent the standard error.

The ANOVA revealed a significant main effect of *IAPS Type* [*F* (2, 92) = 7,600, p = 0,001, power = 0,940]. In particular, the responses were faster with a neutral image than with a pleasant and unpleasant image (558 ms vs. 602 ms vs. 601 ms, respectively). No difference between unpleasant and pleasant pictures was found (602 ms vs. 601 ms; Figure 6).

**Figure 7:**
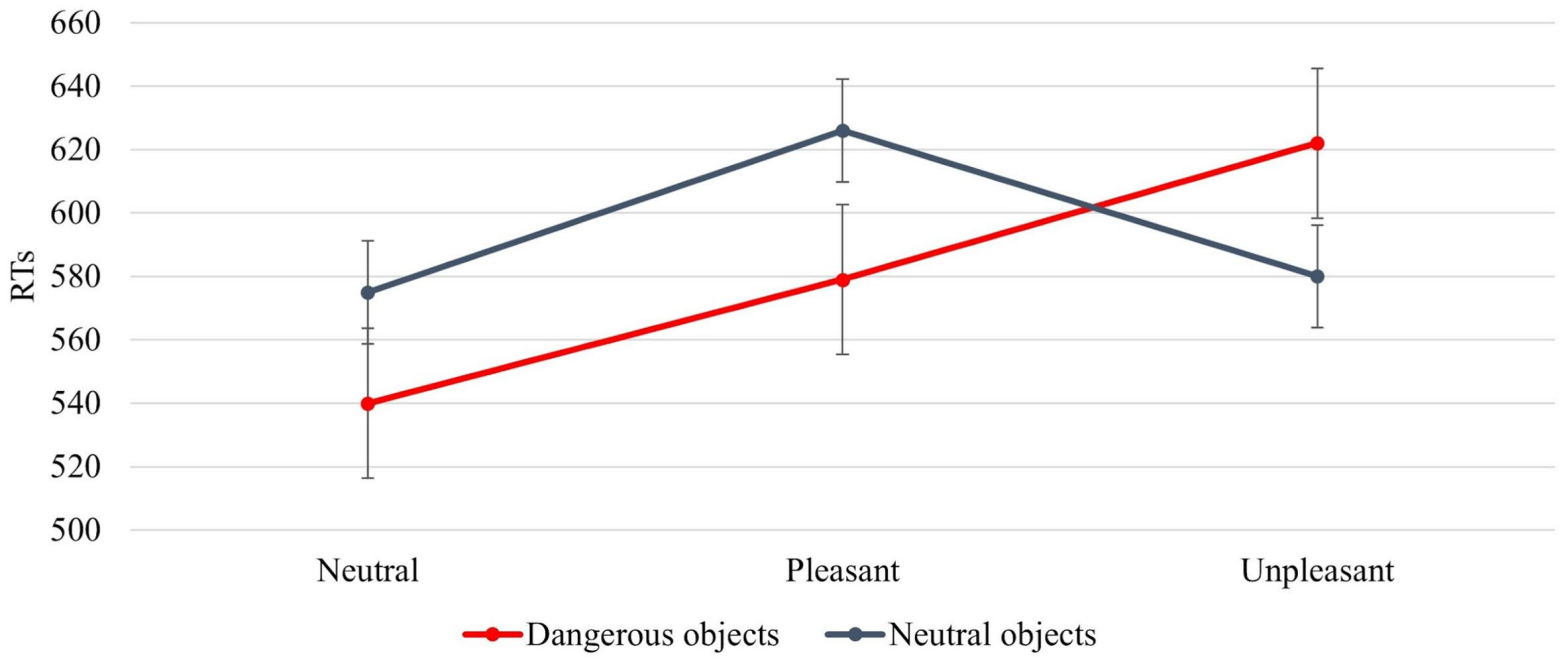
The significant interaction between *Object Dangerousness* and *IAPS Type*. Values are in milliseconds and bars represent the standard error.

The interaction between *IAPS Type* and *Object Dangerousness* was significant [F (2, 92) = 7,649, *p* = 0,001, power = 0,941]. *Post hoc* test revealed that when the object was dangerous, the RTs were faster with a neutral image followed by a pleasant and finally unpleasant image (540 ms, 579 ms, and 622 ms); when the object was neutral, the RTs were faster with a neutral image followed by unpleasant and pleasant image (575 ms, 580 ms, and 626 ms; Figure 7).

No other main effect or interaction was present.

## Discussion

Results confirm our hypothesis about the influence of emotional images on affordances-related motor response. The RTs are faster when the IAPS pictures are neutral than pleasant and unpleasant (Figure 6). This means that despite the pleasant and unpleasant images determining the readiness for action the effect of the cognitive load lowers this readiness (slower RTs). The neutral motivational state guarantees a better readiness to action because it potentiates the action on an object, promoting sufficient attention. This increase may be due to the ability of emotional stimuli to capture, narrow, and hold attention (Ohman et al., 2001; Pourtois et al., 2013). The rapid and efficient selection of emotionally salient or goal-relevant stimuli in the environment is crucial for flexible and adaptive behaviours. When an emotional stimulus is present, the ability to make successive flexible and adaptive behaviours is compromised due to the difficulty of redirecting the attention to other stimuli. The modulation of motor response due to the emotional images is further supported by *Session* main effect. When the *block without IAPS* was the second block presented, the RTs were faster and were influenced by the training effect (Figure 4). The speed due to the effect of training is not present when the *block with IAPS* is the second block presented. In this case, the influence of emotional images is stronger than the training effect.

A significant result is the interaction between *IAPS Type* and *Object dangerousness* (Figure 7). In line with the results discussed above, neutral images guarantee greater attention to stimuli. This is reflected in faster RTs for both neutral and dangerous objects with respect to pleasant and unpleasant images. Therefore, in the case of interaction with objects, not feeling strong emotions favours a better motor performance, facilitating the interaction with potentially dangerous objects. Pleasant and unpleasant images produce two different motivational states. The pleasant images contribute to triggering an approach motivational state supporting the interaction with the object, whereas unpleasant images make an aversive motivational state to avoid the object (Fan and Han, 2008; Han et al., 2008; Decety et al., 2010; Coello et al., 2012; Cole et al., 2013; Anelli et al., 2013b). Figure 7 shows that when the images are pleasant (positive emotion), the interaction with dangerous objects is faster than the interaction with neutral objects, whereas when the images are unpleasant, the interaction is faster with neutral rather than with dangerous objects. This indicates that the aversive motivational state (negative emotion) leads us to pay more attention to the objects around us, preventing us from coming into contact with potentially dangerous objects. In the case of neutral objects, the RTs are faster with unpleasant images (580 ms) than with pleasant images (626 ms). In the first case, RTs are similar when a neutral object is preceded by a neutral image (579 ms).

The literature shows faster RTs for neutral than dangerous objects (Anelli et al., 2012a, 2013b); in our experiment, the RTs are faster for dangerous than neutral objects in the *block without IAPS images* (Figure 5). This result could be explained by considering the different tasks in the previous experiments, the dangerousness of objects has been studied with hand-prime or investigating whether participants were sensitive to differences in the direction of object movement. The harmfulness of an object has never been studied, considering only this feature.

Another interesting result is the significant interaction between *Object Category* and *Response Key* in the *block without IAPS images*. This result shows how the influence of laterality in handedness is maintained. All participants are right-handed, so they are faster at pressing the button with the dominant hand. Since RTs are faster pressing the right button, this affects the object categorization into artifact or natural. This interaction is not present in the *block with IAPS* images: this could mean that, when an emotion is experienced, the influence of laterality in handedness disappears. Normally, the handedness is closely correlated with the emotion categories in the sense that relaxation correlates with left-hand and hostility with right-hand (Kipp and Martin, 2009); but in this study, there is no difference in the presentation of images since the images are presented at the centre of the screen.

### A System-Level Perspective to Understand the Emotional Modulation of Affordances

Our study adds a new and original piece to the theoretical framework supporting the investigations of affordance-related motor response according to a system-level perspective (Caligiore et al., 2010; Thill et al., 2013; de Wit et al., 2017; Osiurak et al., 2017). Until some years ago, object affordances were mainly studied, focusing on single objects. Recent studies have focused on how the context in which different objects are present influences responses to their affordances. For example, it has been shown that the affordances of an object are activated differently depending on whether the object is presented with other objects (Yoon et al., 2010; Borghi et al., 2012; Roux-Sibilon et al., 2018) and in different scenes (Kalénine et al., 2016). In addition, some studies have investigated whether the social context influences affordance activation (for a review, see Borghi, 2018). However, the observer’s motivational state has never been systematically addressed to the best of our knowledge. We explicitly and directly investigated how motivational states influence the affordances-related motor responses elicited by neutral and dangerous objects.

**Figure 8:**
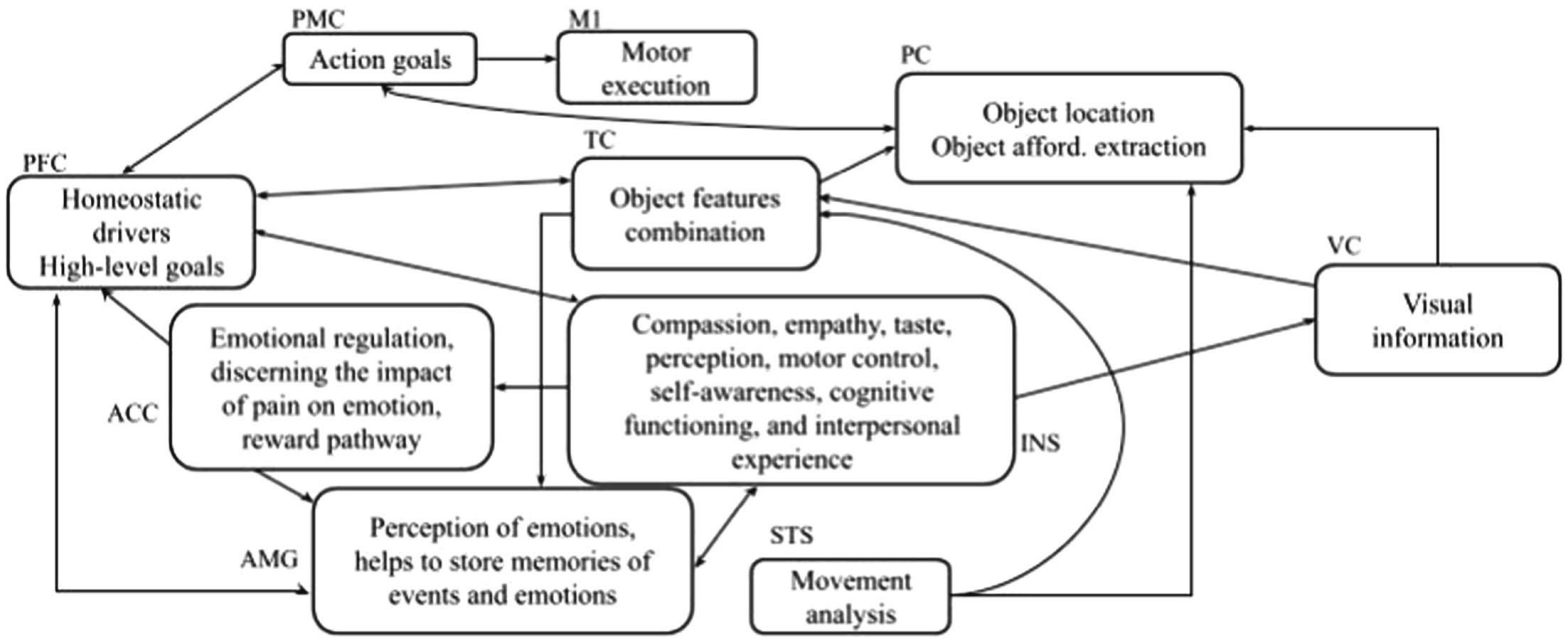
The brain areas and their functions are involved in affordance and emotions. *INS*: insula; *VC*: visual cortex; *M1:* motor area; *ACC:* anterior cingulate cortex; PFC: prefrontal cortex representing dlPFC, vlPFC, dmPFC; *PMC*: premotor cortex; *AMG*: amygdala; *TC*: temporal cortex; *PC:* parietal cortex; and *STC:* superior temporal sulcus.

The system-level approach considers how both the external world (the objects we see) and the inner context (i.e., homeostatic drivers, high-level goals) influence the affordances. Starting from this assumption, the interaction between emotions and affordances can be better explained by considering the brain areas involved in processing the external world and the inner context. Affordances activation involves a cortical–subcortical network, including the parietal sector of the dorsal stream, prefrontal regions as well as basal ganglia and cerebellum (affordance network; Fogassi et al., 2005; Cisek, 2007; Oguro et al., 2009; Caligiore et al., 2010, 2013; Binkofski and Buxbaum, 2013; Thill et al., 2013; Maranesi et al., 2014). However, research has focused on interaction with neutral objects, and what happens when interacting with potentially dangerous objects has not been thoroughly investigated, the only exceptions being from studies on pain (Singer, 2004). This section describes the brain network underlying the emotions and affordances interaction. We start from the literature on affordances, pain, and emotion and use a system-level analysis. This framework supports the interpretation of the results presented here and can suggest the formulation of new experiments in this field. Figure 8 sketches the main brain areas involved in affordances, pain, and emotions.

Within the dorsal neural pathway, the parietal cortex (PC) recognizes the object location, the premotor cortex (PMC) encodes action goals, the superior temporal sulcus (STS) analyses the effects of the observed actions, and the primary motor cortex (M1) supports action execution. The prefrontal cortex areas including the right dorsolateral prefrontal cortex (dlPFC), the left ventrolateral prefrontal cortex (vlPFC), and the dorsomedial prefrontal cortex (dmPFC; all summarized with one abbreviation, PFC, in Figure 8), are strongly connected with Amygdala (AMG) during emotion regulation (Berboth and Morawetz, 2021). PFC forms high-level goals based on two types of information: the outer-world context (based on information received from the associative cortex such as the temporal cortex (TC) within the ventral neural pathway) and the inner-world context (based on information obtained from subcortical areas). PC and TC play complementary roles. PC encodes information about features of objects that are important for guiding manipulation, for example, shape, orientation, three-dimensional aspects, and tactile aspects of objects (Rizzolatti et al., 1998; Sakata et al., 1999; Murata et al., 2000). Based on this evidence, many authors have proposed that PC plays a central role in encoding object affordances (Fagg and Arbib, 1998; Oztop et al., 2004). Various areas within the ventral visual pathway have an activation that responds to highly abstract patterns (e.g., faces, places, and houses) and is rather invariant concerning various aspects of images such as location, orientation, and luminance. Among these areas, TC (ventral occipitotemporal cortex) seems to play a crucial role in object recognition (Grill-Spector and Malach, 2004; Vinberg and Grill-Spector, 2008). The information from the inner-world context is involved in the perception and processing of pain and emotions. The AMG is involved in emotional arousal (LeDoux, 1994; Baxter and Murray, 2002; Phan et al., 2004; Sabatinelli et al., 2005); the insula (INS) is involved in cognitive-emotional processes such as empathy and metacognitive emotional feelings (Critchley et al., 2000a,b, 2002, 2004). The anterior cingulate cortex (ACC) takes care of emotional regulation (Etkin et al., 2011). It is connected with the AMG and INS (ventral ACC) and with PFC (dorsal ACC) making the latter a key station for assigning appropriate control to other brain areas assessing the salience of emotion and motivational information (Bush et al., 2000; Allman et al., 2001). The visual cortex (VC) processes information essential both for object-recognition processes taking place in the ventral pathway and for the sensorimotor transformations guiding action and taking place in the dorsal pathway.

The interaction between the signals conveyed by the dorsal and ventral pathways is critical to understanding the neural mechanisms underlying the results obtained in this article. The RTs, indeed, are strongly related to the time signal processing within the PMC-M1 system that changes according to the different involvement of emotion-related signals conveyed by the PFC (Caligiore et al., 2010; Thill et al., 2013). When the participants feel a neutral motivational state and observe a neutral object (such as in the *block with IAPS images* case, Figure 7) they show the same response speed as when they observe a neutral object without IAPS (Figure 5). This is confirmed by the absence of difference in the RTs for the neutral objects in the *block with IAPS images* and the *block without IAPS images* (575 ms). In both cases, the affordances network must not process emotion-related signals because the emotion is neutral; in this case, the PMC-M1 system processes lower information. When the subject experiences an approach or aversive motivational state, the emotion-related signals affect the affordances network, and the PMC-M1 system processes plus information. This enhanced signal processing produces worse responsiveness (lower RTs; Figure 6).

The literature suggests that neutral objects elicit an affordance effect, such as the approach motivational state, favouring the object’s interaction (Ellis and Tucker, 2000; Phillips and Ward, 2002; Symes et al., 2007). In contrast, dangerous objects elicit an interference/inhibitory effect such as the aversive motivational state aimed to avoid the object (Anelli et al., 2013a; Zhao, 2017). Our results show that this effect depends on the interaction between the object features and the motivational state of the subject. If the subject feels an approach motivational state and observes a dangerous object his/her RTs are similar to those produced when he/she feels an aversive motivational state and observes a neutral object (Figure 7). This is confirmed by the absence of difference between RTs present when the emotional prime is pleasant and the object is dangerous (579 ms) and when the emotional prime is unpleasant and the object is neutral (580 ms). In this case, both emotional and affordance related signals activate similar neural patterns within the dorsal and ventral pathways.

## Limitations

A limitation of the study is that it does not investigate the potentiality of graspable stimuli to elicit an emotion. Only the dangerousness (dangerous vs. neutral) and the category (artifact vs. natural) were studied (Anelli et al., 2012a,b, 2013b). Additionally, no image rating was acquired from the participants, which could have confirmed the effects found based on categorizing the stimuli used according to the IAPS regulatory ratings.

Another limitation is the statistical analysis performed separately for the *block with IAPS images* and *without IAPS images*. The choice is due to the number of trials that constitute each block. The *block without IAPS images* had 96 trials, whereas the *block with IAPS images* included 48 trials. This disparity is due to the number of IAPS images available for the experiment; only 51 images contained no graspable objects used in the study so that they could be used. Furthermore, *a posteriori* power analysis has shown that the number of participants should be around 45–56 participants.

## Conclusion

Traditionally, affordances and emotions have been investigated as two separate processes leading to two different literature threads. In this study, we investigated affordances and emotions as two connected processes. In particular, we studied whether motivational states induced by emotional images influence motor responses and modulate the affordance-related to graspable objects.

Several studies have demonstrated that emotional stimuli may prime the motor system and facilitate action readiness, preparing the body for action (Frijda et al., 1989; Coombes et al., 2009; van Loon et al., 2010). In particular, unpleasant cues activate the defensive system, which facilitates avoidance movements away from the signal (i.e., danger, fear; although anger is one exception; Peterson et al., 2008), whereas pleasant cues activate the appetitive system and facilitate approach movements (i.e., excitement, food, sex; e.g., Chen and Bargh, 1999; Rotteveel and Hans Phaf, 2004). In the case of interaction between affordance and emotion, we found that approach and aversive motivational states slow down the readiness for action, determining slower affordance-motor responses, whereas neutral motivational states guarantee better performance, determining faster affordance-motor responses. Furthermore, experiencing an approach motivational state when relating to dangerous objects could facilitate the interaction with them. Instead, being in an aversive motivational state favours the avoidance of harmful objects.

This study investigated the interaction between affordances and emotions through a system-level approach (Caligiore et al., 2010; Caligiore and Fischer, 2013; Thill et al., 2013) that, based on the underlying brain network, suggested we consider the two processes as mutually dependent rather than as two separate phenomena as usually done so far. Our results and this brain network analysis could be a starting point to devise future electrophysiological works to investigate the neurophysiological mechanisms underlying the affordances-emotions complex interplay.

## Data Availability Statement

The raw data supporting the conclusions of this article will be made available by the authors, without undue reservation.

## Ethics Statement

The studies involving human participants were reviewed and approved by Ethics Committee of the National Research Council of Italy. The patients/participants provided their written informed consent to participate in this study.

## Author Contributions

FG, AB, GB, and DC: conceptualization, data curation, investigation, methodology, validation, and writing - review and editing. FG: formal analysis, software, visualization, and writing - original draft. GB: funding acquisition. DC: project administration and supervision. FG and AB: resource. All authors contributed to the article and approved the submitted version.

## Funding

This research was supported by European Union’s Horizon 2020 Research and Innovation program under grant agreement no. 952095 project IM-TWIN from Intrinsic Motivations to Transitional Wearable INtelligent companions for autism spectrum disorder (https://cordis.europa.eu/project/id/952095/it) and by the Advanced School in Artificial Intelligence (www.as-ai.org). The authors declare that this study received funding from AI2Life s.r.l. (www.ai2life.com). The funder was not involved in the study design, collection, analysis, interpretation of data, the writing of this article or the decision to submit it for publication.

## Conflict of Interest

GB and DC are employed by the company AI2Life s.r.l.

The remaining authors declare that the research was conducted in the absence of any commercial or financial relationships that could be construed as a potential conflict of interest.

## Publisher’s Note

All claims expressed in this article are solely those of the authors and do not necessarily represent those of their affiliated organizations, or those of the publisher, the editors and the reviewers. Any product that may be evaluated in this article, or claim that may be made by its manufacturer, is not guaranteed or endorsed by the publisher.

## Footnotes

^1^The library numbers for IAPS pictures (Lang et al., 2008) used in this study are: neutral: 2020, 2107, 2200, 2210, 2215, 2271, 2280, 2357, 2385, 2441, 2493, 2499, 7183, 7184, 7237, 7249, 9070; pleasant: 4311, 4608, 4643, 4651, 4652, 4656, 4660, 4664, 4668, 4676, 4683, 4687, 4689, 4693, 4697, 4698, 8470; unpleasant: 2352.2, 3000, 3010, 3051, 3059, 3060, 3061, 3062, 3071, 3101, 3130, 3131, 3160, 3168, 3170, 3185, 3225

^2^The library numbers for IAPS pictures (Lang et al., 2008) used in online survey was: neutral: 2020, 2107, 2190, 2200, 2210, 2214, 2215, 2271, 2280, 2357, 2385, 2397, 2440, 2441, 2493, 2499, 2512, 2570, 2620, 4500, 7183, 7184, 7237, 7249, 7493, 7495, 9070, 9260; pleasant: 4290, 4311, 4608, 4611, 4643, 4651, 4652, 4656, 4658, 4660, 4664, 4666, 4668, 4672, 4676, 4680, 4681, 4683, 4687, 4689, 4690, 4693, 4694, 4695, 4697, 4698, 4800, 5470, 8185, 8470; unpleasant: 2352.2, 2799, 3000, 3010, 3030, 3051, 3059, 3060, 3061, 3062, 3063, 3064, 3068, 3069, 3071, 3100, 3101, 3102, 3110, 3120, 3130, 3131, 3140, 3150, 3160, 3168, 3170, 3185, 3195, 3225, 3230, 9253, 9412
